# A Study on Preparation and Property Evaluations of Composites Consisting of TPU/Triclosan Membranes and Tencel^®^/LMPET Nonwoven Fabrics

**DOI:** 10.3390/polym14122514

**Published:** 2022-06-20

**Authors:** Bing-Chiuan Shiu, Po-Wen Hsu, Jian-Hong Lin, Ling-Fang Chien, Jia-Horng Lin, Ching-Wen Lou

**Affiliations:** 1 College of Material and Chemical Engineering, Minjiang University, Fuzhou 350108, China; bcshiu@mju.edu.cn (B.-C.S.); j0928451554@gmail.com (P.-W.H.); baron.lin69@gmail.com (J.-H.L.); 2 Fujian Key Laboratory of Novel Functional Fibers and Materials, Minjiang University, Fuzhou 350108, China; 3 Laboratory of Fiber Application and Manufacturing, Department of Fiber and Composite Materials, Feng Chia University, Taichung 40724, Taiwan; m0801831@mail.fcu.edu.tw; 4 Department of Medical Research, China Medical University Hospital, China Medical University, Taichung 40402, Taiwan; 5 Advanced Medical Care and Protection Technology Research Center, College of Textile and Clothing, Qingdao University, Qingdao 266071, China; 6 Innovation Platform of Intelligent and Energy-Saving Textiles, School of Textile Science and Engineering, Tiangong University, Tianjin 300387, China; 7 School of Chinese Medicine, China Medical University, Taichung 40402, Taiwan; 8 Department of Bioinformatics and Medical Engineering, Asia University, Taichung 413305, Taiwan

**Keywords:** low melting point polyester (LMPET), triclosan, Tencel^®^ nonwoven fabrics, antibacterial TPU

## Abstract

This study investigated eco-friendly antibacterial medical protective clothing via the nonwoven process and characteristic evaluations. Firstly, Tencel^®^ fibers and low melting point polyester (LMPET) fibers (re-sliced and granulated from recycled PET bottles) were mixed at different ratios and then needle punched at diverse needle rolling depths. The influences of manufacturing parameters on the Tencel^®^/LMPET nonwoven fabrics were examined in terms of mechanical properties, water vapor transmission rate, and stiffness. Next, Tencel^®^/LMPET nonwoven fabrics were combined with thermoplastic polyurethane (TPU)/Triclosan antibacterial membranes that contained different contents of triclosan using melt processing technology. The resulting Tencel^®^/LMPET/TPU/Triclosan composites were characterized via different measurements; an optimal bursting strength of 86.86 N, an optimal horizontal tensile strength of 41.90 N, and an optimal stiffness along the MD and CD of 8.60 cm were recorded. Furthermore, the Tencel^®^/LMPET/TPU/Triclosan composites exhibited a distinct inhibition zone in the antibacterial measurement, and the hydrostatic pressure met the requirements of the EN 14126:2003 and GB 19082-200 disposable medical protective gear test standards.

## 1. Introduction

Tencel^®^ is the commercial title of Lyocell fibers that are cellulous fibers made by solvent spinning wood pulp using N-Methylmorpholine-N-oxide (NMMO) as the solvent. What Lyocell fibers employ is a cellulose fiber technique that does not require a chemical reaction [1]. When dissolved in NMMO, cellulose fibers do not break down. NMMO and polyhydroxy (-OH) jointly generate hydrogen bonds that can dissolve cellulose, during which the closed production process transforms cellulose into an electrospinning solution. The electrospinning solution becomes cellulose fibers with the help of the dry-jet wet spinning technology. Specifically, over 99% of NMMO can be recycled and reused without jeopardizing the environment, and it can naturally decompose, featuring a clean production and decomposition, both of which are eco-friendly [2,3,4]. Products consisting of Lyocell fibers have noticeable qualities of high strength, high purity, and high hygroscopicity while appearing fluffy and drape due to the crimped feature. Moreover, they can retain the intrinsic chemical properties in a wet state. Tencel^®^ fibers are natural fibers with a minor impact on the environment as they can be biodegraded, offering a solution to the waste fabrics from disposable products [5]. Furthermore, Tencel^®^ fibers also account for a considerable market share of ready-made clothes, and they are suitable for high-end shirts, underwear, and casual wear. Additionally, they can directly absorb perspiration from users into the fiber molecules, leaving no residual sweat on the skin, and preventing the growth of bacteria and the discomfort rendered by moisture and heat. Similarly, Tencel^®^ fibers also have unique advantages when applied to livelihood supplies, sanitary articles, industrial cloth, medical bandages, and adult/infant diapers, featuring a broad range of applications [6,7,8].

Low melting point polyester (LMPET) staple fibers have been commonly used in global markets and are preferred by many large-scale yarn spinning factories since LMPET fibers possess a low melting point, indicating an equivalently good adhesion quality. LMPET fibers are primarily used to replace resin or viscose [9,10]. In addition to a better quality of nonwoven products, the use of LMPET fibers significantly addresses air pollution, water pollution, and the like, all of which are disadvantages to using resin adhesives [11]. Resembling the chemical structure of polyester, LMPET fibers are made by copolymerization and modification, containing phthalic acid and ethylene glycol through esterification and condensation polymerization. Moreover, amorphous LMPET fibers have the highest yield and are the most frequently used fibers with dimensional stability, heat resistance, and wear- and weather-resistant properties [12], the characteristics of which differ from those of conventional polyester fibers. The melting point of LMPET fibers is 110 °C, and they have a thermal shrinkage of 5% when in a free state. By contrast, polypropylene and polyamide exhibit flow deformation after being melted, and the cortical structure exhibits thermoplasticity. As a result, the melted skin of LMPET fibers bonds the particles or fibers; however, the melted state is not reversible and indicates a high heat shrinkage. Hence, LMPET fibers can substitute polypropylene and polyamide hot melt adhesives and are thus used for the thermal consolidation process, strengthening the formability and improving mechanical properties [13,14]. Recycled polyester fibers have a lower melting point (7–12 °C) and lower crystallinity as compared to virgin polyester fibers. The tenacity of recycled polyester fibers is lower (~15%) as compared to that of their virgin polyester counterparts, which can be attributed to the thermal degradation of the polymer during secondary melting [15]. LMPET made from recycled PET bottles has decreased mechanical properties due to the decrease in various temperature indicators and mechanical properties after secondary melting [16,17]. When LMPET comes from recycled PET bottles, the temperature index drops to the same point as the thermal bonding temperature of TPU [18,19].

Thermoplastic polyurethane (TPU), also known as polyurethane, is a polymer composed of polyisocyanate, polyether, polyester, macromolecule polyol, small molecule polyols, polyamine, water, a chain extender, and a cross-linking agent [20,21]. TPU has an abrasion resistance that is five times greater than that of natural rubber, while its good tear properties, transparency, and elasticity suggest that TPU is a polymer that shares similar characteristics with rubber and plastic [22,23]. With excellent manufacturing stability, and comprehensive physical and chemical properties, TPU can substitute polyvinyl chloride (PVC) and polyurethane (PU) to satisfy the eco-friendly purpose to fit the requirements of national defense, medical hygiene, sporting goods, electronic appliances, and the food industry [24,25]. TPU membranes are made of TPU pellets through a special process. There is well-developed TPU waterproof/breathable clothing which includes special TPU films for shoe material lamination, special films specially designed for silicone breast pads, and TPU elastic ribbons developed in the 20th century. In effect, TPU is essentially a linear structural polymer and can be applied using the same technique and equipment as for thermoplastic plastics, e.g., injection, extruding, blow molding, and calendaring. In sum, TPU is particularly suitable for mass production of medium- and small-sized components.

Triclosan (TCS) is also known as Dichlorophenoxychlorophenol, with the chemical formula of C_12_H_7_Cl_3_O_2_, and is an efficient antibacterial agent and disinfectant. TCS appears resistant to biodegradation and is commonly a white or grayish crystal powder that emits a phenolic odor [26]. TCS is not water-soluble but easily dissolves in lye and organic solvents. TCS is a broad-spectrum antibacterial agent used against hepatitis B virus and staphylococcus. Everyday necessities grant an upper limit of triclosan of 0.3%; as such, it is commonly used in soap, toothpaste, and other chemical products [27,28]. The sterilization mechanism is that TCS adsorbs and then penetrates the cell walls of bacteria to interact with the lipids and proteins of the cytoplasm, thereby deteriorating the protein so as to kill the bacteria [29,30]. More TCS is added to medicated mouthwash when used in the treatment for Candida albicans, gingivitis, or mouth ulcers. Moreover, TCS can also be used as a plastic additive in the production of antibacterial trash bags and the like [31].

In this study, Tencel^®^ fibers and LMPET fibers were blended at different ratios (LMPET comes from recycled PET bottles), and the blends underwent the nonwoven process with different needle punch depths in order to form Tencel^®^/LMPET nonwoven matrices. Next, a single-screw granulator was used to blend TPU pellets with diverse contents of TCS powders, forming TPU/Triclosan antibacterial membranes. Finally, Tencel^®^/LMPET nonwoven matrices and TPU/Triclosan antibacterial membranes were combined to form eco-friendly antibacterial composites used in protective clothing. The composite nonwoven fabrics were tested for stiffness, water vapor transmission rate, scanning electron microscopic observation, antibacterial efficacy, and mechanical properties, thereby determining the optimal Tencel^®^/LMPET/TPU/Triclosan antibacterial composites for protective clothing matrices. Taking advantage of the characteristics of the lower melting point of recycled PET bottles, LMPET was compounded with TPU/Triclosan by a hot pressing process without using adhesives. Whether LMPET or Tencel^®^ fibers are recycled or regenerated fibers, this research proposes such a concept in the hope that nonwoven fabrics used in medical protective clothing during the time of COVID-19 can be improved.

## 2. Materials and Methods

### 2.1. Materials

The Tencel^®^ staple fibers, previously named Lyocell (Haoshen Fiber Technology Co., Ltd., Jiangsu, China), had a length of 51 mm and a fineness of 1.7 dtex. The low melting point polyester (LMPET) fibers (re-sliced and granulated from recycled PET bottles) (Huvis Co., Ltd., Incheon, Korea) had a length of 64 mm, a fineness of 4D, and a melting point of 110 °C. Thermoplastic polyurethanes (TPU, # HV-7280EB, Zhanyu Polyurethane Co., Ltd., Shanghai, China) had a melting index of 32 g/10 min (190 °C/8.7 kg). Triclosan (Wuqi Biotechnology Co., Ltd., Henan, China) had a molecular weight of 300. The needle type for the needle punch was M332G53017 (Groz-Beckert Co., Ltd., Baden-Wurttemberg, Germany). *Staphylococcus aureus* (*S. aureus*) meets ATCC25923 and *Escherichia coli* (*E*. *coli*) meets ATCC25922. *S. aureus* and *E*. *coil* were purchased from the Food Industry Research and Development Institute, Shanghai, China.

### 2.2. Preparation of Tencel^®^/LMPET Nonwoven Fabrics

**Figure 1:**
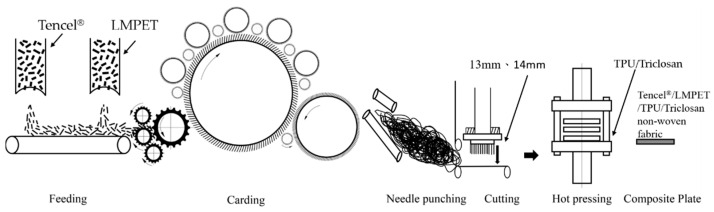
Diagram illustrating the manufacturing process of Tencel^®^/LMPET nonwoven fabrics, adapted from [32], SAGE Publications, 2014.

A porcupine opener was used to scatter Tencel^®^ and LMPET fibers separately, and the fibers were then mixed at blending ratios of 100/0, 90/10, 80/20, 70/30, and 60/40 wt% using a needle curtain. Afterwards, the Tencel^®^/LMPET blends became fluffy due to the breaking-and-opening process by a cotton comber, a cylinder, and a doffer. The blended fibers were carded to form an aligned fiber mesh that was then transmitted to the top screen for lay-up formation. Passing through the shake screen of a reciprocating traverse forming machine, the aligned Tencel^®^/LMPET fiber mesh was laminated over the bottom screen and then needle bonded via a needle punching machine, forming Tencel^®^/LMPET nonwoven fabrics (Figure 1). The needle punch parameters included a speed of 200 needle/min and a depth of 13 mm or 14 mm. Finally, the nonwoven fabrics and TPU/Triclosan underwent roller thermoforming at 160 °C at a speed of 7 rpm/min with a two-wheel embossing machine.

### 2.3. Preparation of Tencel^®^/LMPET/TPU Matrices for Antibacterial Membranes

**Figure 2:**
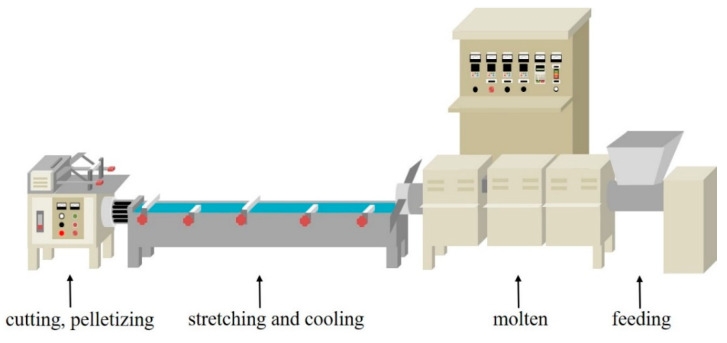
Schematic diagram of the melt blending process for TPU pellets and TCS powders via a single-screw granulator.

In the production of TPU/Triclosan antibacterial membranes, the content of triclosan was 0, 0.01, 0.05, 0.1, and 0.2 wt% (Figure 2). To start with, TPU pellets and triclosan powders were melt-blended at successive temperatures of 160, 170, and then 180 °C with a single-screw granulator, after which the blends were placed in a cooling bath to shape the TPU/Triclosan pellets. Finally, the pellets were dissolved in a TPU dissolution reaction tank and subsequently formed into TPU/Triclosan membranes with a thickness of 20 μm using a laminator. The Tencel^®^/LMPET nonwoven fabric and TPU/Triclosan composite heat pressing temperature was 160 °C.

### 2.4. Characterizations

**Figure 3:**
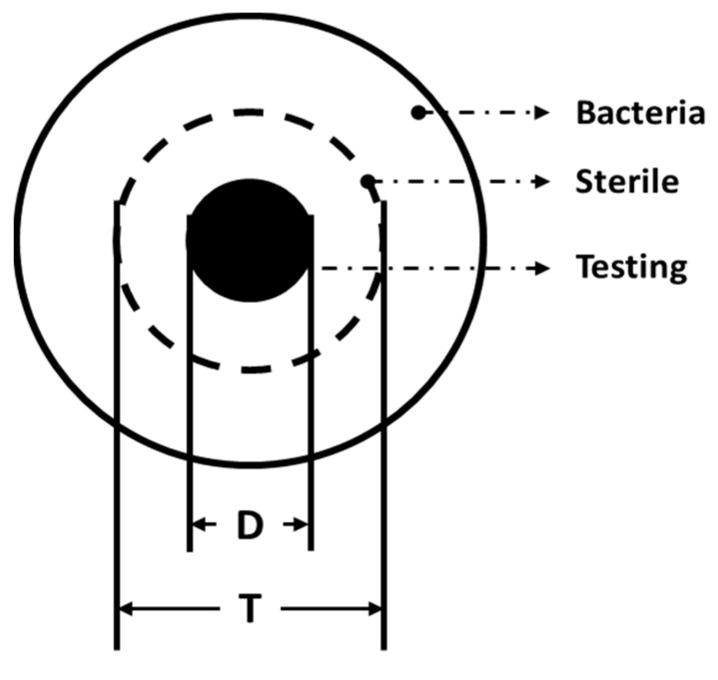
Diagram of the inhibition zone.

The air permeability of the fabrics was measured using an air permeability tester (TEXTEST FX 3300-IV, Libero Technology Co., Ltd., Guangzhou, China) as specified in the ASTM D737-04 test standard. The tearing strength of samples was measured using a universal strength testing machine (HT-2402, Hong Ta Co., Ltd., Taiwan, China) as specified in the CNS 12915 test standard; five samples of 150 mm × 75 mm for each specification were taken along the machine direction (MD) and the cross-machine direction (CD) for an average. The bursting strength of samples (130 mm × 130 mm) was measured at a test rate of 10 mm/min using a universal strength testing machine (HT-2402, Hong Ta Co., Ltd., Taiwan) as specified in the CNS 12915 test standard. The tensile strength of the samples (180 mm × 250 mm) was measured at a test rate of 305 ± 13 mm/min using a universal strength testing machine (HT-2402, Hong Ta Co., Ltd., Taiwan) as specified in the CNS 12915 test standard. The distance between clamps was 7.6 cm, and six samples for each specification were taken along the MD and CD. Afterwards, the average, standard deviation, and coefficient of variation were computed accordingly. The stiffness test was conducted using the cantilever method as specified in the CNS 12915 test standard, and the stiffness is presented in centimeters. The antibacterial measurement was conducted as specified in the JIS1902-2002 test standard with *S. aureus* (ATCC 25923) and *E. coli* (ATCC 25922). The presence and the size of the inhibition zone (Figure 3) were observed and determined using Equation (1) [33]. The material structure, morphology, and element analysis of the fabricated materials were characterized by field-emission scanning electron microscopy (Hitachi SEM 4800, Tokyo, Japan).

Inhibition zone = T − D(1)

where D means the diameter of the circular sample (i.e., 6-mm), and T shows whether the sample exhibits an inhibition zone. When (T − D) > 0 is true, it means that the sample has antibacterial efficacy.

## 3. Results and Discussion

### 3.1. Tearing Strength and Stiffness of Tencel^®^/LMPET Nonwoven Fabrics

**Figure 4:**
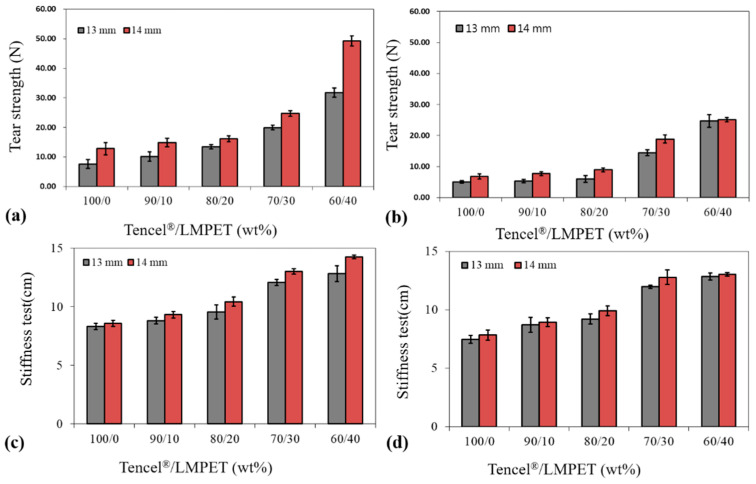
Tear strength along the (**a**) CD and (**b**) MD as well as stiffness along the (**c**) CD and (**d**) MD of Tencel^®^/LMPET nonwoven fabrics.

Figure 4a,b show the tensile strength along the CD and MD of Tencel^®^/LMPET nonwoven fabrics. The nonwoven process involves a needle punch that contributes mechanical reinforcement of the resulting nonwoven fabrics as the fibers are entangled and bonded in the fiber mesh. Based on the test results, a rise in the LPET fiber content had a positive influence on the tensile strength of Tencel^®^/LMPET nonwoven fabrics. In particular, with a blending ratio of 60/40 wt%, Tencel^®^/LMPET nonwoven fabrics exhibited a maximal tensile strength of 52.3 N, the result of which can be attributed to the presence of LMPET fibers. When hot-pressed, the sheath of LMPET fibers melted to form thermal bonding points; the more LMPET fibers, the more thermal bonding points, which in turn mechanically reinforces Tencel^®^/LMPET nonwoven fabrics to a certain degree. On the other hand, Figure 4c,d indicate that Tencel^®^/LMPET nonwoven fabrics showed a decreasing trend in stiffness when composed of more Tencel^®^ fibers. Without any special treatment, nonwoven fabrics consisting of LMPET fibers exhibit less comfort than those consisting of natural fibers. Hence, the presence of LMPET fibers has a negative influence on the air permeability and stiffness of Tencel^®^/LMPET nonwoven fabrics. Contrastingly, the subsequent lamination process and hot pressing also adversely affect the stiffness, which illustrates the uncomfortable texture of current medical protective clothing. In this study, the Tencel^®^/LMPET ratio of 50/50 wt% was also employed, but the resulting nonwoven fabrics were composed of an excessive amount of LMPET fibers, which means that there were equally too many LMPET fibers forming too many thermal bonding points. Eventually, the stiffness of the nonwoven fabrics was compromised too much, and the 50/50 group was thus excluded.

### 3.2. Air Permeability of Tencel^®^/LMPET Nonwoven Fabrics

**Figure 5:**
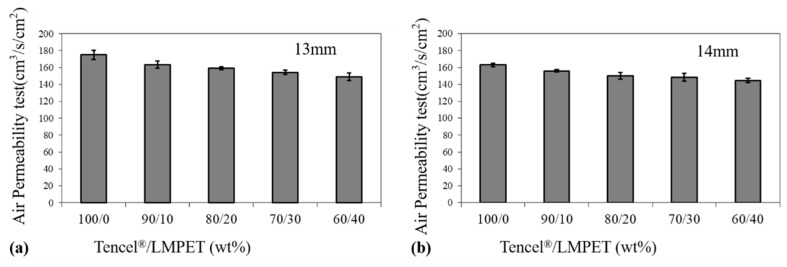
Air permeability of Tencel^®^/LMPET nonwoven fabrics that were needle punched at a depth of (**a**) 13 mm and (**b**) 14 mm in relation to the Tencel^®^/LMPET blending ratios.

The air permeability of nonwoven fabrics is dependent on the size and quantity of the voids among fibers. The voids are correlated with the fiber traits, fabric structure, fabric thickness, and fabric finishing. The nonwoven air permeability mechanism means that, being isolated by the fabric, the air with a higher pressure passes through the fabric from one specified side to the opposite side. Figure 5 compares the air permeability of Tencel^®^/LMPET nonwoven fabrics when they were produced using a needle punch depth of 13 mm and 14 mm, and the air permeability fell in the range of 144.75~175 cm^3^/s/cm^2^. Tencel^®^ fibers have a smooth and sleek surface and feature excellent moisture repelling, air permeability, and cooling properties. Hence, Tencel^®^/LMPET nonwoven fabrics showed a higher air permeability when consisting of more Tencel^®^ fibers.

Moreover, a higher needle punch depth also showed a negative influence on the air permeability of the Tencel^®^/LMPET nonwoven fabrics because the fibers were entangled to a greater extent when they were needle punched by barbed fibers at a greater depth. By comparison, the 60/40 blending ratio provided nonwoven fabrics with a marginally lower strength than the 100/0 blending ratio, yet the 60/40 group still outperformed the other groups with different blending ratios in terms of mechanical properties. As a result, the optimal Tencel^®^/LMPET nonwoven fabrics were produced with a blending ratio of 60/40 wt% and a needle punch depth of 14 mm, and this specified group of nonwoven fabrics was combined with the antibacterial TPU/triclosan membranes afterwards.

### 3.3. Bursting Strength of Tencel^®^/LMPET/TPU/Triclosan Composites

**Figure 6:**
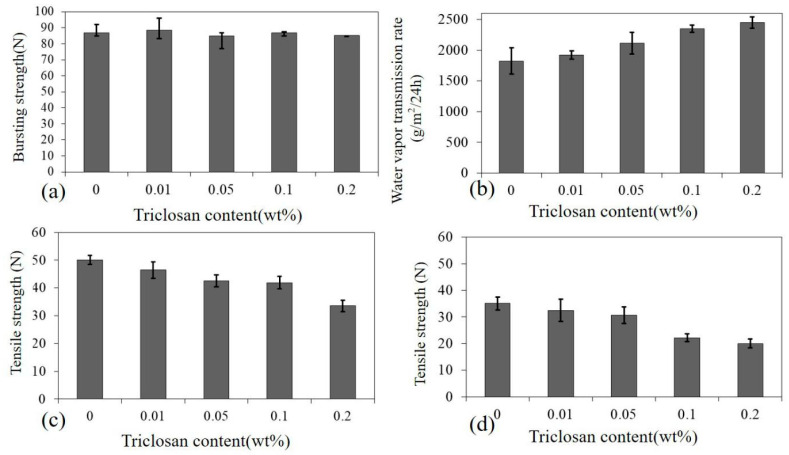
(**a**) Bursting strength, (**b**) water vapor transmission, and tensile strength along the (**c**) MD and (**d**) CD of Tencel^®^/LMPET nonwoven fabrics (60/40).

Figure 6a shows that the Tencel^®^/LMPET/TPU/Triclosan composites had an optimal bursting strength of 83 ± 7 N, while Figure 6b shows the test results of water vapor transmission that indicates how efficiently the composites release moisture and sweat vapor to keep wearers comfortable and dry without locking in heat around the skin. Based on EN 14126:2003 and GB 19082-2003 (specifications for verification of disposable one-piece medical protective clothing), the water vapor transmission of Tencel^®^/LMPET/TPU/Triclosan composites was measured and found to be better than the requirement of the test standard (1500 g/m^2^/24 h) [34,35,36,37]. Furthermore, Figure 6c shows that the tensile strength along the MD was 53.2 N for the composites without triclosan. When the composites were composed of a greater amount of triclosan, the tensile strength showed a declining trend. With 0.2 wt% of triclosan, the tensile strength along the CD of the composites was 33.5 N. Figure 6d compares the influence of the MD and the CD on the tensile strength of the Tencel^®^/LMPET/TPU/Triclosan composites. The tensile strength along the MD was lower and was only 20.1 N. Similarly, with a rise in the triclosan content, the tensile strength of the composites also decreased. Due to the presence of triclosan, the membranes contained more impurities, which in turn jeopardized the tensile strength.

### 3.4. SEM Observation of Tencel^®^/LMPET/TPU/Triclosan Composites

**Figure 7:**
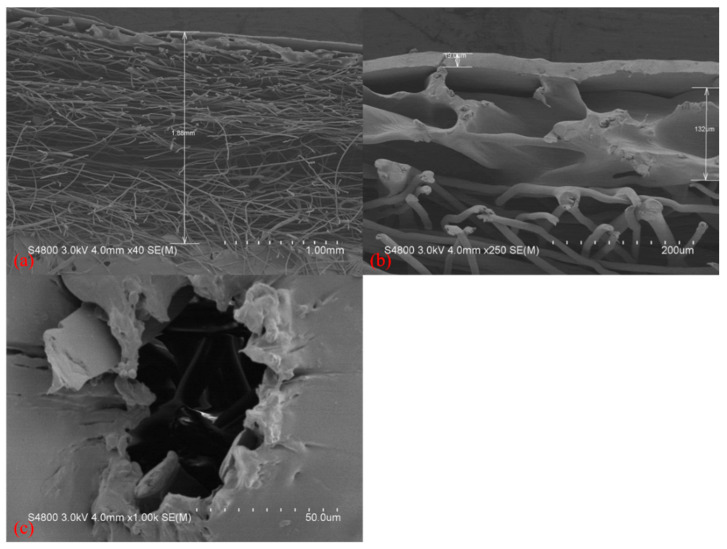
SEM images of Tencel^®^/LMPET/TPU/Triclosan composites at (**a**) 40×, (**b**) 250× magnification showing sectional observation and (**c**) after bursting strength measurement: 1000×.

Figure 7a is the SEM image showing the trimmed Tencel^®^/LMPET/TPU/Triclosan composites with a thickness of 1.88 mm, while the original thickness of the composites was 3 mm. Figure 7b shows that the TPU/Triclosan antibacterial membranes had a thickness of 20 ± 0.12 µm, which changed marginally after undergoing the lamination process. Figure 7c shows the SEM image of fractured composites after the bursting strength test. The bursting strength performance of the TPU/Triclosan antibacterial membranes correlated with the presence of triclosan at diverse contents except for 0.1%. Consecutive cracking is absent in the resulting TPU/Triclosan antibacterial membranes that consist of 0.1 wt% of triclosan.

### 3.5. Antibacterial Efficacy of Tencel^®^/LMPET/TPU/Triclosan Composites

**Figure 8:**
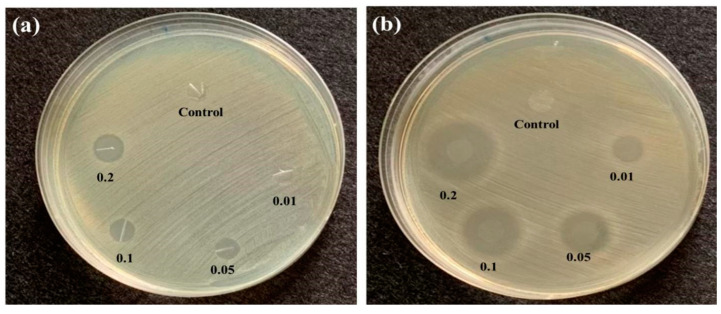
Inhibition zone of antibacterial TPU/Triclosan membranes against (**a**) *E. coli* and (**b**) *S. aureus* in relation to the triclosan content (wt%).

In this study, triclosan was used as an antibacterial agent, and *S. aureus* (Gram-positive bacteria) and *E. coli* (Gram-negative bacteria) were used in the qualitative antimicrobial testing. The antibacterial measurement results are shown in Table 1. Figure 8 indicates that the antibacterial efficacy was proportional to the triclosan content. Specifically, the inhibition zone against *S. aureus* was 5.9 ± 0.3 mm when the antibacterial membranes consisted of 0.1 wt% of triclosan, showing a stabilized trend. In Figure 8b, the antibacterial membranes exhibit greater antibacterial efficacy against *S. aureus* than *E. coli*. Triclosan and silver ions share the same antibacterial mechanism, and the surface of both items carries positive charges that can draw the negative charges of the bacteria, thereby damaging the cell walls of the bacteria as well as preventing the fatty acids from synthesizing. Subsequently, the growth and function of biological cells are hampered, which eventually causes the death of the bacteria. In addition, the most significant difference between Gram-positive bacteria and Gram-negative bacteria is the thickness of the cell walls, which means that *S. aureus* possesses a lower thickness and is vulnerable to the penetration of triclosan. Hence, TPU/Triclosan membranes can prevent the synthesis of fatty acids inside the cytoplasm, attaining antibacterial efficacy or even achieving a sterilization effect.

### 3.6. Stiffness and Water Resistance of Tencel^®^/LMPET/TPU/Triclosan Composites

**Figure 9:**
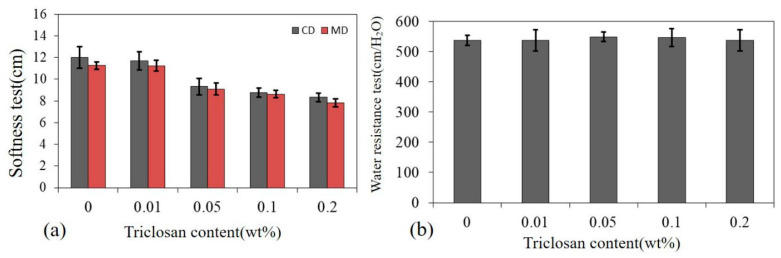
(**a**) Stiffness and (**b**) water resistance of Tencel^®^/LMPET/TPU/Triclosan composites.

Figure 9a shows the stiffness of Tencel^®^/LMPET/TPU/Triclosan composites in relation to the triclosan content. During the combination, the seepage of TPU/Triclosan reduced the friction level of the fluffy Tencel^®^/LMPET nonwoven fabrics, improving the stiffness concurrently. The seepage also expelled the air in the voids of the nonwoven fabrics, resulting in a greater adhesion level between the Tencel^®^/LMPET nonwoven fabrics and the TPU/Triclosan antibacterial membranes. Regardless of whether it was along the MD or CD, the stiffness of the Tencel^®^/LMPET/TPU/Triclosan composites improved. Figure 9b shows the water resistance measurement (JISL 1092 B) of the Tencel^®^/LMPET/TPU/Triclosan composites as specified in EN 14126:2003 and GB 19082-2003 (specifications for verification of disposable one-piece medical protective clothing) [37]. Samples with a water pressure resistance as high as 140 cm/H_2_O meet the requirements of the test standards. Regardless of the triclosan content, all of the Tencel^®^/LMPET/TPU/Triclosan composites are qualified to provide a water resistance of 537–549 cm/H_2_O, since TPU molecular chains contain smaller-sized molecular arrangement holes and therefore possess the waterproof feature. At room temperature, TPU membranes have waterproof properties without being jeopardized by water or moisture and provide the composites with an advantage that they can be used in a high-humidity environments or in contact with water. Regarding EN 14126:2003 and GB 19082-2003, it is stipulated that the strength of materials in key parts of protective clothing is not less than 45 N, and the data in this study are all greater than 45 N. The standard stipulates that the hydrostatic pressure should not be lower than 1.67 kPa (17 cm/H_2_O). This study found a value of 537–549 cm/H_2_O, which depends on the superior performance of TPU.

## 4. Conclusions

Overall, Tencel^®^/LMPET nonwoven fabrics were optimal when the blending ratio was 60/40 wt% and the needle punch depth was 14 mm. Comparing to a needle punch depth of 13 mm, the needle punch depth of 14 mm improved the tearing strength, bursting strength, and tensile strength by 65%, 27%, and 11%, respectively. In addition, a needle punch depth of 15 mm broke the needles, whereas a needle punch depth of 12 mm failed to entangle the fibers effectively; thus, the optimal needle punch depth is 14 mm. As for TPU/Triclosan antibacterial membranes with a thickness of 20 μm containing triclosan (0, 0.01, 0.05, 0.1, and 0.2 wt%), only 0.1 wt% of triclosan helped to generate a distinctive inhibition zone and provided the membranes with antibacterial efficacy; however, 0.2 wt% of triclosan showed an adverse effect on the mechanical properties. Furthermore, with a trivial amount of triclosan, TPU membranes acquired antibacterial efficacy while improving diverse properties, which suggests that the incorporation of triclosan is one of the valuable additive parameters. Compared with the standard as shown in Table 2 below, the tensile strength in this study needs to be improved in the CD direction, and the moisture permeability also needs to be improved. Tencel^®^ has lower strength than polyester, but better comfort, and LMPET uses recycled PET bottle slices, meaning the strength will be decreased. All other indicators meet the standard requirements. One of the reasons for the lack of strength is that no adhesive was used. The low melting point of LMPET was used for bonding with nonwoven fabrics through hot-pressed bonding points.

## Figures and Tables

**Table 1:** Inhibition zone (mm) of antibacterial TPU/Triclosan membranes against *E. coli* and *S. aureus*.

Triclosan Content (wt%)	*E. coli*	*S. aureus*
	Inhibition Zone (mm)	
Triclosan 0	0	0
Triclosan 0.01	0.6	1.3
Triclosan 0.05	1.0	3.4
Triclosan 0.1	1.3	5.9
Triclosan 0.2	2.0	7.4

**Table 2:** The results of this study are compared with the EN 14126:2003 and GB 19082-2003 standards.

	Standard Specification	Results of This Study
Tensile strength	MD and CD ≥ 45 N	MD 53.2 N, CD 33.5 N
Bursting strength	≥45 N	83 N
Strong seam	≥40 N	-----------------
Tear strength	≥20 N	53.5 N
Moisture permeability	≥2500 g/m^2^.24 h	2350 g/m^2^.24 h
Hydrostatic pressure	≥50 cm H_2_O	537–549 cm/H_2_O
Grade penetration	≥1 g	-----------------
Microbial indicators	not checked	obvious inhibition zone

## Data Availability

This study did not report any data.

## References

[1] Jiang X., Bai Y., Chen X., Liu W. (2020). A review on raw materials, commercial production and properties of lyocell fiber. J. Bioresour. Bioprod..

[2] Perepelkin K.E. (2007). Lyocell fibres based on direct dissolution of cellulose in N-methylmorpholine N-oxide: Development and prospects. Fibre Chem..

[3] Zhang S., Chen C., Duan C., Hu H., Li H., Li J., Liu Y., Ma X., Stavik J., Ni Y. (2018). Regenerated cellulose by the Lyocell process, a brief review of the process and properties. BioResources.

[4] Wang S., Lu A., Zhang L. (2016). Recent advances in regenerated cellulose materials. Prog. Polym. Sci..

[5] Shen L., Worrell E., Patel M.K. (2010). Environmental impact assessment of man-made cellulose fibres. Resour. Conserv. Recycl..

[6] Basit A., Latif W., Baig S.A., Afzal A. (2018). The Mechanical and Comfort Properties of Sustainable Blended Fabrics of Bamboo with Cotton and Regenerated Fibers. Cloth. Text. Res. J..

[7] Asmiza N.A.N., Nasir S.H. (2021). A Review on Mechanical and Comfort Properties of Medical Bandages from Different Material and Finishing. Prog. Eng. Appl. Technol..

[8] Kim H.-A. (2021). Moisture Vapor Permeability and Thermal Wear Comfort of Ecofriendly Fiber-Embedded Woven Fabrics for High-Performance Clothing. Materials.

[9] Lin J.-H., Hsu P.-W., Huang C.-H., Lai M.-F., Shiu B.-C., Lou C.-W. (2022). A Study on Carbon Fiber Composites with Low-Melting-Point Polyester Nonwoven Fabric Reinforcement: A Highly Effective Electromagnetic Wave Shield Textile Material. Polymers.

[10] Pang K., Kotek R., Tonelli A. (2006). Review of conventional and novel polymerization processes for polyesters. Prog. Polym. Sci..

[11] Patti A., Cicala G., Acierno D. (2020). Eco-sustainability of the Textile Production: Waste Recovery and Current Recycling in the Composites World. Polymers.

[12] Hsieh J.-C., Li J.-H., Huang C.-H., Lou C.-W., Lin J.-H. (2017). Statistical analyses for tensile properties of nonwoven geotextiles at different ambient environmental temperatures. J. Ind. Text..

[13] Douka A., Vouyiouka S., Papaspyridi L.-M., Papaspyrides C.D. (2018). A review on enzymatic polymerization to produce polycondensation polymers: The case of aliphatic polyesters, polyamides and polyesteramides. Prog. Polym. Sci..

[14] Kucuk M., Korkmaz Y. (2015). Sound absorption properties of bilayered nonwoven composites. Fibers Polym..

[15] Majumdar A., Shukla S., Singh A.A., Arora S. (2020). Circular fashion: Properties of fabrics made from mechanically recycled poly-ethylene terephthalate (PET) bottles. Resour. Conserv. Recycl..

[16] Pudack C., Stepanski M., Fässler P. (2020). PET Recycling—Contributions of Crystallization to Sustainability. Chem. Ing. Tech..

[17] Welle F. (2011). Twenty years of PET bottle to bottle recycling—An overview. Resour. Conserv. Recycl..

[18] Jiang J., Liu F., Yang X., Xiong Z., Liu H., Xu D., Zhai W. (2021). Evolution of ordered structure of TPU in high-elastic state and their influences on the autoclave foaming of TPU and inter-bead bonding of expanded TPU beads. Polymer.

[19] Yao Y., Xiao M., Liu W. (2021). A Short Review on Self-Healing Thermoplastic Polyurethanes. Macromol. Chem. Phys..

[20] Boubakri A., Haddar N., Elleuch K., Bienvenu Y. (2010). Impact of aging conditions on mechanical properties of thermoplastic polyurethane. Mater. Des..

[21] Pattanayak A., Jana S.C. (2005). Properties of bulk-polymerized thermoplastic polyurethane nanocomposites. Polymer.

[22] El-Shekeil Y.A., Sapuan S.M., Abdan K., Zainudin E.S. (2012). Influence of fiber content on the mechanical and thermal properties of Kenaf fiber reinforced thermoplastic polyurethane composites. Mater. Des..

[23] Tan J., Ding Y.M., He X.T., Liu Y., An Y., Yang W.M. (2008). Abrasion resistance of thermoplastic polyurethane materials blended with ethylene-propylene-diene monomer rubber. J. Appl. Polym. Sci..

[24] Dimonie D., Coserea R.M., Singurel G., Zaharia C., Darie R.N., Pop S.F. (2009). Rheological properties of polyvinyl chloride—Thermoplastic polyurethane blends. Mater. Plast..

[25] Parnell S., Min K. (2005). Reactive blending of thermoplastic polyurethane in situ with poly(vinyl chloride). Polym. Eng. Sci..

[26] Dann A.B., Hontela A. (2011). Triclosan: Environmental exposure, toxicity and mechanisms of action. J. Appl. Toxicol..

[27] Caswell K.J., Long S.K. (2015). The expanding role of managed care in the Medicaid program: Implications for health care access, use, and expenditures for nonelderly adults. INQUIRY J. Health Care Organ. Provis. Financ..

[28] Montaseri H., Forbes P.B. (2016). A review of monitoring methods for triclosan and its occurrence in aquatic environments. TrAC Trends Anal. Chem..

[29] Li M., He Y., Sun J., Li J., Bai J., Zhang C. (2019). Chronic Exposure to an Environmentally Relevant Triclosan Concentration Induces Persistent Triclosan Resistance but Reversible Antibiotic Tolerance in *Escherichia coli*. Environ. Sci. Technol..

[30] Shrestha P., Zhang Y., Chen W.-J., Wong T.-Y. (2020). Triclosan: Antimicrobial mechanisms, antibiotics interactions, clinical applications, and human health. J. Environ. Sci. Health C Toxicol. Carcinog..

[31] Gavilanes-Martínez M.A., Coral-Garzón A., Cáceres D.H., García A.M. (2021). Antifungal activity of boric acid, triclosan and zinc oxide against different clinically relevant Candida species. Mycoses.

[32] Sathishkumar T., Naveen J., Satheeshkumar S. (2014). Hybrid fiber reinforced polymer composites—A review. J. Reinf. Plast. Compos..

[33] Lou C.W., Wu Z.H., Lin J.H. (2014). Processing Technique and Property Evaluations of Chitosan Dressings. Adv. Mater. Res..

[34] Nguyen T.-T., Tran D.-T., Bui T.-A. (2020). A Study on Designing a Machine for Testing the Resistance to Wet Bacterial Penetration of Medical Protective Clothing Based on the British Standard EN 14126: 2003. Proceedings of the 2nd Annual International Conference on Material, Machines and Methods for Sustainable Development (MMMS2020).

[35] Laing R.M. (2008). Protection Provided by Clothing and Textiles Against Potential Hazards in the Operating Theatre. Int. J. Occup. Saf. Ergon..

[36] Kim H.-A. (2021). Water Repellency/Proof/Vapor Permeability Characteristics of Coated and Laminated Breathable Fabrics for Outdoor Clothing. Coatings.

[37] Ahmed T., Ogulata R.T., Bozok S.S. (2021). Silver nanoparticles against SARS-CoV-2 and its potential application in medical protective clothing—A review. J. Text. Inst..

